# Detection of myeloma cell-derived microvesicles: a tool to monitor multiple myeloma load

**DOI:** 10.1186/s40164-023-00392-4

**Published:** 2023-03-06

**Authors:** Zhao-Yun Liu, Nan-Hao Meng, Pan-Pan Cao, Feng-Ping Peng, Jing-Yi Luo, Hao Wang, Feng-Juan Jiang, Jin Lu, Rong Fu

**Affiliations:** 1 Department of Hematology, Tianjin Medical University General Hospital, 154 Anshan Street, Heping District, Tianjin, 300052 People’s Republic of China; 2 Peking University People’s Hospital, Peking University Institute of Hematology, National Clinical Research Center for Hematologic Disease, Beijing, 100044 People’s Republic of China; 3 Innovative Center of Hematology, Soochow University, Suzhou, 215031 People’s Republic of China

## Abstract

The persistence of tumor load in multiple myeloma (MM) lead to relapse in patients achieving complete remission (CR). Appropriate and effective methods of myeloma tumor load monitoring are important for guiding clinical management. This study aimed to clarify the value of microvesicles in monitoring MM tumor load. Microvesicles in bone marrow and peripheral blood were isolated by differential ultracentrifugation and detected by flow cytometry. Western blotting was applied to assess myosin light chain phosphorylation levels. Flow cytometry to detect Ps^+^CD41a^−^, Ps^+^CD41a^−^CD138^+^, Ps^+^CD41a^−^BCMA^+^ microvesicles from bone marrow can be used to predict myeloma burden, furthermore, Ps^+^CD41a^−^ microvesicles may as a potential index to MRD test. Mechanistically, the releasing of microvesicles from MM cell was regulated by Pim-2 Kinase via Phosphorylation of MLC-2 protein.

**Supplementary Information:**

The online version contains supplementary material available at 10.1186/s40164-023-00392-4.

**Dear editor,**

Multiple myeloma (MM) is a malignant clonal disease of the plasma cells in bone marrow (BM) [1]. In recent years, MM tumor load decreased dramatically after effective therapy [2]. However, residual tumor load often leads to relapse in remission patients. It is important for appropriate methods for tumor load detection to guide clinical management [3, 4].

The existing tools widely used to monitor MM tumor load include multicolor flow cytometry, next-generation sequencing, imaging assessments such as MRI and PET-CT [5, 6]. However, due to the focal distribution of MM cells, the test results may not accurately reflect the tumor load; at the same time, with treatment and deep remission, the sensitivity of MRD detection has become more essential [7]. It is known that microvesicles carry various markers which can reflect their origin; thus, it is feasible to monitor MM load by detecting microvesicles from MM cell via flow cytometry [8, 9].

First, we isolated bone marrow supernatant microvesicles from MM patients by differential centrifugation and verified microvesicles morphology by electron microscopy (Additional file 1: Fig S1). The number of microvesicles was detected by flow cytometry (Additional file 1: Fig S2). The number of CD41a^−^Phosphatidylserine^+^ (Ps^+^) microvesicles from BM was significantly higher in newly diagnosed multiple myeloma (NDMM) (n = 49) than in healthy donors (HDs) (n = 34) and in complete remission (CR) (n = 40) (p < 0.001) (Fig. 1A, Additional file 2: Table S2). The number of CD138, BCMA, and CD319 positive (specific markers for MM cell) non-platelet-derived microvesicles was analyzed, the number of Ps^+^CD41a^−^CD138^+^, Ps^+^CD41a^−^BCMA^+^, Ps^+^CD41a^−^CD319^+^microvesicles from BM in NDMM was significantly higher than that in CR and HDs (p < 0.001) (Fig. 1B, Additional file 2: Table S2). Further, the number of BM microvesicles from 11 matched newly diagnosed and treated MM patients who achieved CR were detected, we found the number of CD41a^−^Ps^+^, Ps^+^CD41a^−^CD138^+^, Ps^+^CD41a^−^BCMA^+^, Ps^+^CD41a^−^CD319^+^microvesicles was significantly higher in the initial treatment group than in the remission group after treatment (p < 0.001) (Fig. 1C). Correlation analysis showed that the number of BM microvesicles labeled CD41a^−^Ps^+^, Ps^+^CD41a^−^CD138^+^, Ps^+^CD41a^−^BCMA^+^, Ps^+^CD41a^−^ CD319^+^ was positively correlated with the number of β2-MG(p < 0.001) and plasma cells (p < 0.001) in bone marrow smear (Fig. 1D). ROC curve was analyzed to distinguish the patients with NDMM from those without. The AUC of CD41a^−^Ps^+^, Ps^+^CD41a^−^CD138^+^, Ps^+^CD41a^−^BCMA^+^, and Ps^+^CD41a^−^CD319^+^ microvesicles from BM were 0.9519, 0.9666, 0.9408, 0.8890 (p < 0.001), the sensitivity was 99.00%, 99.00%, 90.91%, 88.64%, and the specificity was 94.44%, 85.29%, 85.29%, 85.29%, respectively (Fig. 1E left, Table 1). To distinguish NDMM from CR patients, the AUC was 0.7351, 0.7705, 0.7966, 0.7165 (p < 0.001), the sensitivity was 89.36%, 90.91%, 86.36%, 65.91%, and the specificity was 60.00%, 57.50%, 70.00%, and 70.00%, respectively (Fig. 1E right, Table 1). These results showed that CD138, BCMA, CD319 single-positive non-platelet-derived microvesicles can potentially reflect the MM tumour load.

**Fig. 1:**
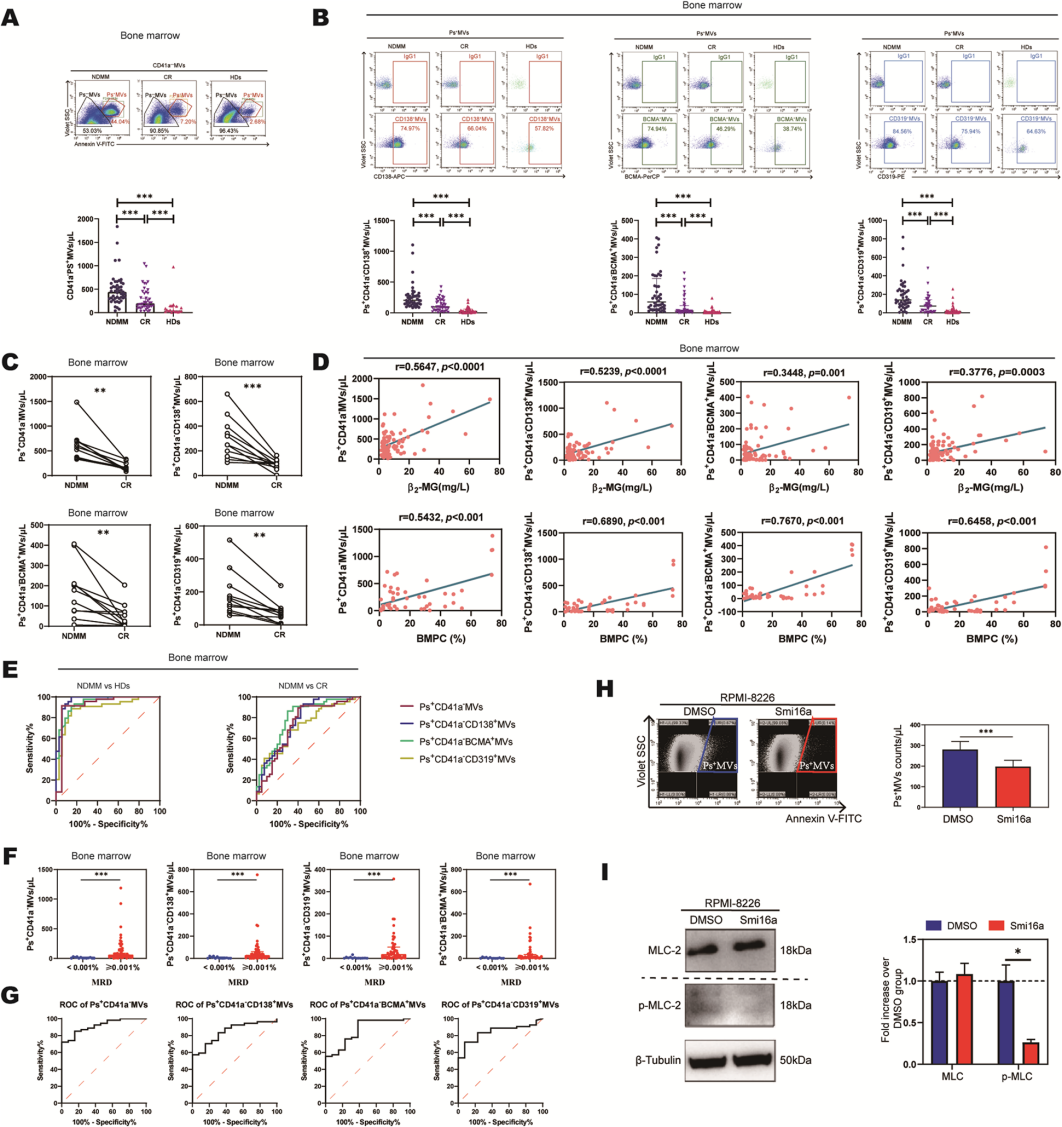
The value of microvesicles in bone marrow in monitoring MM tumor load. **A** The number of CD41a^−^Ps^+^ microvesicles from BM was significantly higher in NDMM compared with in CR and HDs. **B** The number of Ps^+^CD41a^−^CD138^+^, Ps^+^CD41a^−^BCMA^+^, Ps^+^CD41a^−^CD319^+^ microvesicles from BM was significantly higher in NDMM compared with in CR and HDs. **C** The number of CD41a^−^Ps^+^, Ps^+^CD41a^−^CD138^+^, Ps^+^CD41a^−^BCMA^+^, Ps^+^CD41a^−^CD319^+^ microvesicles from BM was significantly higher in the initial treatment group (NDMM) than in the remission group (CR) after treatment in 11 matched patients. **D** The number of BM microvesicles labeled CD41a^−^Ps^+^, Ps^+^CD41a^−^CD138^+^, Ps^+^CD41a^−^BCMA^+^, Ps^+^CD41a^−^ CD319^+^ was positively correlated with the number of β2-MG and plasma cells in bone marrow smear.** E** ROC curve was analyzed Ps^+^CD41a^−^CD138^+^, Ps^+^CD41a^−^BCMA^+^, Ps^+^CD41a^−^CD319^+^ microvesicles from BM to distinguish the NDMM patients from HDs (left) and CR patients (right). **F** The number of BM microvesicles labeled CD41a^−^Ps^+^, Ps^+^CD41a^−^CD138^+^, Ps^+^CD41a^−^BCMA^+^, Ps^+^CD41a^−^CD319^+^ was significantly higher in MRD ( +) group compared with in MRD (−) group. **G** ROC curve was analyzed CD41a^−^Ps^+^, Ps^+^CD41a^−^CD138^+^, Ps^+^CD41a^−^BCMA^+^, Ps^+^CD41a^−^CD319^+^ microvesicles from BM to distinguish the MRD ( +) patients from MRD (−) patients. The number of Ps^+^CD41a^−^ microvesicles has high accuracy (AUC > 0.9). **H** CD41a^**−**^Ps^+^ microvesicles were significantly reduced in the presence of Smi16a. **I** p-MLC-2 protein values decreased in the presence of Smi16a

**Table 1:** The AUC values of ROC curves

	AUC (95%CI)	*P* Value	Optimal		
			Cutoff Points, μL	Sensitivity, %	Specificity, %
NDMM vs HDs (BM)					
CD41a-Ps +	0.9519(0.8598–0.9900)	< 0.001	193.20	99.00	94.44
Ps + CD41a-CD138 +	0.9666(0.9275–0.9900)	< 0.001	73.03	99.00	85.29
Ps + CD41a-BCMA +	0.9408(0.8906–0.9911)	< 0.001	11.78	90.91	85.29
Ps + CD41a-CD319 +	0.8890(0.8119–0.9662)	< 0.001	44.22	88.64	85.29
Ps + CD41a-CD138 + BCMA +	0.7961(0.6941–0.8981)	< 0.001	6.61	79.55	70.59
Ps + CD41a-CD138 + CD319 +	0.7874(0.6871–0.8877)	< 0.001	48.67	65.91	82.35
Ps + CD41a-BCMA + CD319 +	0.7059(0.5873–0.8245)	0.002	11.75	59.09	79.41
NDMM vs HDs (PB)					
CD41a-Ps +	0.8596(0.7681–0.9511)	< 0.001	27.02	81.82	76.67
Ps + CD41a-CD138 +	0.8636(0.7769–0.9504)	< 0.001	5.105	93.94	68.75
Ps + CD41a-BCMA +	0.7325(0.6065–0.8584)	0.001	2.365	87.88	59.38
Ps + CD41a-CD319 +	0.7784(0.6643–0.8925)	< 0.001	5.495	81.82	71.88
Ps + CD41a-CD138 + BCMA +	0.6795(0.5487–0.8102)	0.01	0.890	59.38	75.76
Ps + CD41a-CD138 + CD319 +	0.7476(0.6301–0.8652)	< 0.001	3.340	57.58	84.38
Ps + CD41a-BCMA + CD319 +	0.8016(0.6946–0.9086)	< 0.001	2.020	69.70	84.38
NDMM vs CR (BM)					
CD41a-Ps +	0.7351(0.6258–0.8444)	< 0.001	223.60	89.36	60.00
Ps + CD41a-CD138 +	0.7705(0.6689–0.8720)	< 0.001	101.90	90.91	57.50
Ps + CD41a-BCMA +	0.7966(0.6992–0.8939)	< 0.001	15.70	86.36	70.00
Ps + CD41a-CD319 +	0.7165(0.6070–0.8260)	< 0.001	104.40	65.91	70.00
Ps + CD41a-CD138 + BCMA +	0.5858(0.4634–0.7082)	0.18	108.30	20.45	97.50
Ps + CD41a-CD138 + CD319 +	0.6463(0.5286–0.7604)	0.02	102.90	36.36	95.00
Ps + CD41a-BCMA + CD319 +	0.6011(0.4758–0.7237)	0.11	101.3	34.56	97.00
NDMM vs CR (PB)					
CD41a-Ps +	0.8013(0.6917–0.9210)	< 0.001	25.78	84.85	74.07
Ps + CD41a-CD138 +	0.7168(0.5776–0.8476)	0.004	5.180	55.17	90.91
Ps + CD41a-BCMA +	0.7168(0.5843–0.8494)	0.003	1.720	51.72	93.94
Ps + CD41a-CD319 +	0.6855(0.5465–0.8244)	0.010	3.950	44.83	93.94
Ps + CD41a-CD138 + BCMA +	0.6390(0.4961–0.7819)	0.060	0.890	55.17	75.76
Ps + CD41a-CD138 + CD319 +	0.6473(0.5098–0.7848)	0.050	2.230	58.62	69.70
Ps + CD41a-BCMA + CD319 +	0.7048(0.5721–0.8375)	0.006	1.990	75.86	69.70
MRD (−) vs MRD ( +) (BM)					
CD41a-Ps +	0.9160(0.8471–0.9848)	< 0.001	33.22	72.22	92.31
Ps + CD41a-CD138 +	0.8433(0.7427–0.9439)	< 0.001	22.25	55.65	84.62
Ps + CD41a-BCMA +	0.8590(0.7530–0.9649)	< 0.001	1.165	98.15	61.54
Ps + CD41a-CD319 +	0.8412(0.7436–0.9387)	< 0.001	6.305	72.22	92.31

Then, we analyzed the CD138, BCMA, CD319 double-positive non-platelet-derived microvesicles. We found the number of Ps^+^CD41a^−^CD138^+^CD319^+^ microvesicles from BM were significantly high in NDMM than those in CR and HDs (p < 0.05) (Additional file 1: Fig S3A, Additional file 2: Table S2). In 11 matched patients, the number of these microvesicles were also significantly high in NDMM than in treated MM patients who achieved CR (Additional file 1: Fig S3B). The correlation analysis showed that the number of these microvesicles was also positively correlated with the number of β2-MG (p < 0.001), and plasma cells (p < 0.001) in bone marrow smear (Additional file 1: Fig S3C). ROC curve was analyzed Ps^+^CD41a^−^CD138^+^BCMA^+^, Ps^+^CD41a^−^CD138^+^CD319^+^, Ps^+^CD41a^−^BCMA^+^CD319^+^ microvesicles from BM to distinguish the patients with NDMM from those without. The AUC of these microvesicles were 0.7961, 0.7874, 0.7059 (p < 0.05), the sensitivity was 79.55%, 65.91%, 59.09%, and the specificity was 70.59%, 82.35%, 79.41%, respectively (Additional file 1: Fig S3D left, Table 1). To distinguish NDMM from CR patients, the AUC was 0.5858, 0.6463, 0.6011 (p < 0.05), the sensitivity was 20.45%, 36.36%, 34.56%, and the specificity was 97.50%, 95.00%, 97.00% respectively (Additional file 1: Fig S3D right, Table 1). These results indicated that CD138, BCMA, CD319 double-positive non-platelet-derived microvesicles can potentially predict the MM tumor load but show no advantage than CD138, BCMA, CD319 single-positive non-platelet-derived microvesicles.

Next, we explore the detecting role of microvesicles from peripheral blood (PB) to monitor MM tumor load as a non-invasive operation. The peripheral blood microvesicles were analyzed. We found the number of CD41a^−^Ps^+^, Ps^+^CD41a^−^CD138^+^, Ps^+^CD41a^−^BCMA^+^, Ps^+^CD41a^−^CD319^+^ microvesicles from PB was significantly higher in NDMM (n = 33) than in HDs (n = 32) and in CR (n = 29) (p < 0.001) (Fig S4A, Additional file 2: Table S3). Furthermore, the number of CD41a^−^Ps^+^, Ps^+^CD41a^−^CD138^+^, Ps^+^CD41a^−^BCMA^+^, Ps^+^CD41a^−^CD319^+^ microvesicles from 11 matched newly diagnosed and treated MM patients who achieved CR was also compared, which found that the initial treatment group was significantly higher than the remission group (p < 0.001) (Additional file 1: Fig S4B). Correlation analysis showed only the number of CD41a^−^Ps^+^ microvesicles was positively correlated with the number of β2-MG (p < 0.001) (Additional file 1: Fig S4C). ROC curve was analyzed PB microvesicles labeled with CD41a^−^Ps^+^, Ps^+^CD41a^−^CD138^+^, Ps^+^CD41a^−^BCMA^+^, Ps^+^CD41a^−^CD319^+^ to distinguish NDMM patients from HDs. The AUC of these microvesicles were 0.8596, 0.8636, 0.7325, 0.7784 (p < 0.05), the sensitivity was 81.82%, 93.94%, 87.88%, 81.82%, and the specificity was 76.67%, 68.75%, 59.38%, 71.88%, respectively (Additional file 1: Fig S4D, Table 1). To distinguish NDMM from CR patients. The AUC was 0.8013, 0.7168, 0.7168, 0.6855 (p < 0.05), and the sensitivity was 84.85%, 55.17%, 51.72%, 44.83%, and the specificity was 74.07%, 90.91%, 93.94%, 93.94% respectively (Additional file 1: Fig S4D, Table 1). Similarly, to further study the clinical value of CD138, BCMA and CD319 double positive microvesicles from BM in predicting MM tumor load, we analyzed the number of Ps^+^CD138^+^BCMA^+^, Ps^+^CD138^+^CD319^+^, Ps^+^BCMA^+^CD319^+^ non-platelet-derived microvesicles. ROC curves were analyzed these microvesicles to distinguish NDMM from HDs, the AUC of microvesicles labeled with Ps^+^CD41a^−^CD138^+^BCMA^+^, Ps^+^CD41a^−^CD138^+^CD319^+^, Ps^+^CD41a^−^BCMA^+^CD319^+^ were 0.6795, 0.7476, 0.8016 (p < 0.05), the sensitivity was 59.38%, 57.58%, 69.70%, and the specificity was 75.76%, 84.38%, 84.38% respectively (Additional file 1: Fig S4E, Table 1); to distinguish NDMM from CR, the AUC was 0.6390, 0.6473, 0.7048 (p < 0.05), the sensitivity was 55.17%, 58.62%, 75.86%, and the specificity was 75.76%, 69.70%, 69.70% respectively (Additional file 1: Fig S4E, Table 1). These results indicated microvesicles from peripheral blood as potential index to predict the tumor burden in MM patients.

From all above AUC data from both BM and PB, the number of Ps^+^CD41a^−^ (0.9519), Ps^+^CD41a^−^CD138^+^ (0.9666), Ps^+^CD41a^−^BCMA^+^ (0.9408) microvesicles from BM of MM patients to monitor MM tumor load has high accuracy since AUC value above 0.9 (Additional file 3).

To further assess the reliability of microvesicles from BM to monitor tumor load, we compared the number of Ps^+^CD41a^−^, Ps^+^CD41a^−^CD138^+^, Ps^+^CD41a^−^BCMA^+^, Ps^+^CD41a^−^CD319^+^ microvesicles in bone marrow of 50 MM patients who underwent MRD testing (Euro-flow method). The MRD positive group (n = 37) was significantly higher than the MRD negative group (n = 13)(p < 0.001) (Fig. 1F, Additional file 2: Table S4). The AUC of these microvesicles were 0.9160, 08,433, 0.8590, 08,412 (p < 0.05), the sensitivity was 72.22%, 55.65%, 98.15%, 72.22%, and the specificity was 92.31%, 84.62%, 61.54%, 92.31%, respectively (Fig. 1G, Table 1). It is further suggested that the number of Ps^+^CD41a^−^ microvesicles in bone marrow may be potential indicators for MM patient MRD test according AUC data.

Phosphorylation of myosin light chain (MLC) is an important mechanism for the release of microvesicles from cells, and previous studies have suggested that RabGTPase-activating proteins such as RhoA and ARF6 proteins mediate microvesicles formation and release by affecting serine phosphorylation at position 19 on the myosin light chain [10, 11]. Is it possible that Pim-2 kinase, as a member of serine/threonine family, which may mediate the release of MM cell microvesicles by affecting the phosphorylation of MLC. Our study showed that smi16a (a Pim-2 kinase inhibitor) [12] significantly reduced the release of microvesicles from MM cell compared with PBS (197.19 ± 31.302 vs. 280.42 ± 39.346, p < 0.001) (Fig. 1H), and the level of p-MCL-2 in Smi16a group was significantly lower than that in PBS group (Fig. 1I). It indicated that Pim-2 kinase can increase MLC phosphorylation and regulate microvesicles release.

In conclusion, Ps^+^CD41a^−^, Ps^+^CD41a^−^CD138^+^, Ps^+^CD41a^−^BCMA^+^microvesicles from bone marrow as a potential role to monitor MM load, and Ps^+^CD41a^−^ microvesicles from bone marrow may as a potential index to MRD test. Mechanically, Pim-2 involved in regulating microvesicles releasing through p-MCL-2. This liquid biopsy can solve the false negative of bone marrow aspiration to some extent (Additional file 1: Fig S5).

## Supplementary Information

**Additional file 1.** Additional figures 1–5.

**Additional file 2.** Additional tables 1–6.

**Additional file 3.** Materials and methods.

## Data Availability

All datasets that the conclusions of the paper rely on are available to readers and deposited in publicly available repositories.

## Abbreviations

MM: Multiple myeloma
CR: Complete remission
BM: Bone marrow
Ps: Phosphatidylserine
NDMM: Newly diagnosed multiple myeloma
HDs: Healthy donors
MLC: Myosin light chain
