# Supplementary material for: Detection of myeloma cell-derived microvesicles: a tool to monitor multiple myeloma load
Source: Exp Hematol Oncol. 2023 Mar 6;12:26. doi: 10.1186/s40164-023-00392-4 (PMC9987071; doi:10.1186/s40164-023-00392-4)
Supplement: Supplementary file 1 — Additional file 1. Additional figures 1–5. [file 40164_2023_392_MOESM1_ESM.docx]

**Additional file1**

**Fig S1**


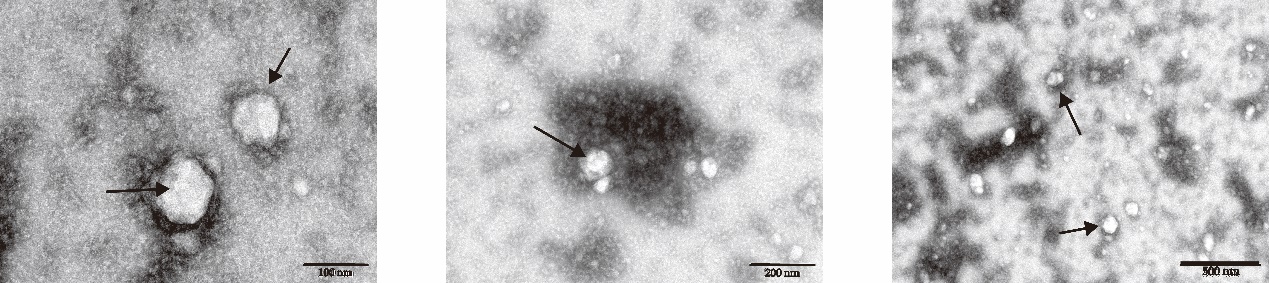


Identification of microvesicles by TEM. TEM scanning showed the images of microvesicles derived from MM cells.

**Fig S2**


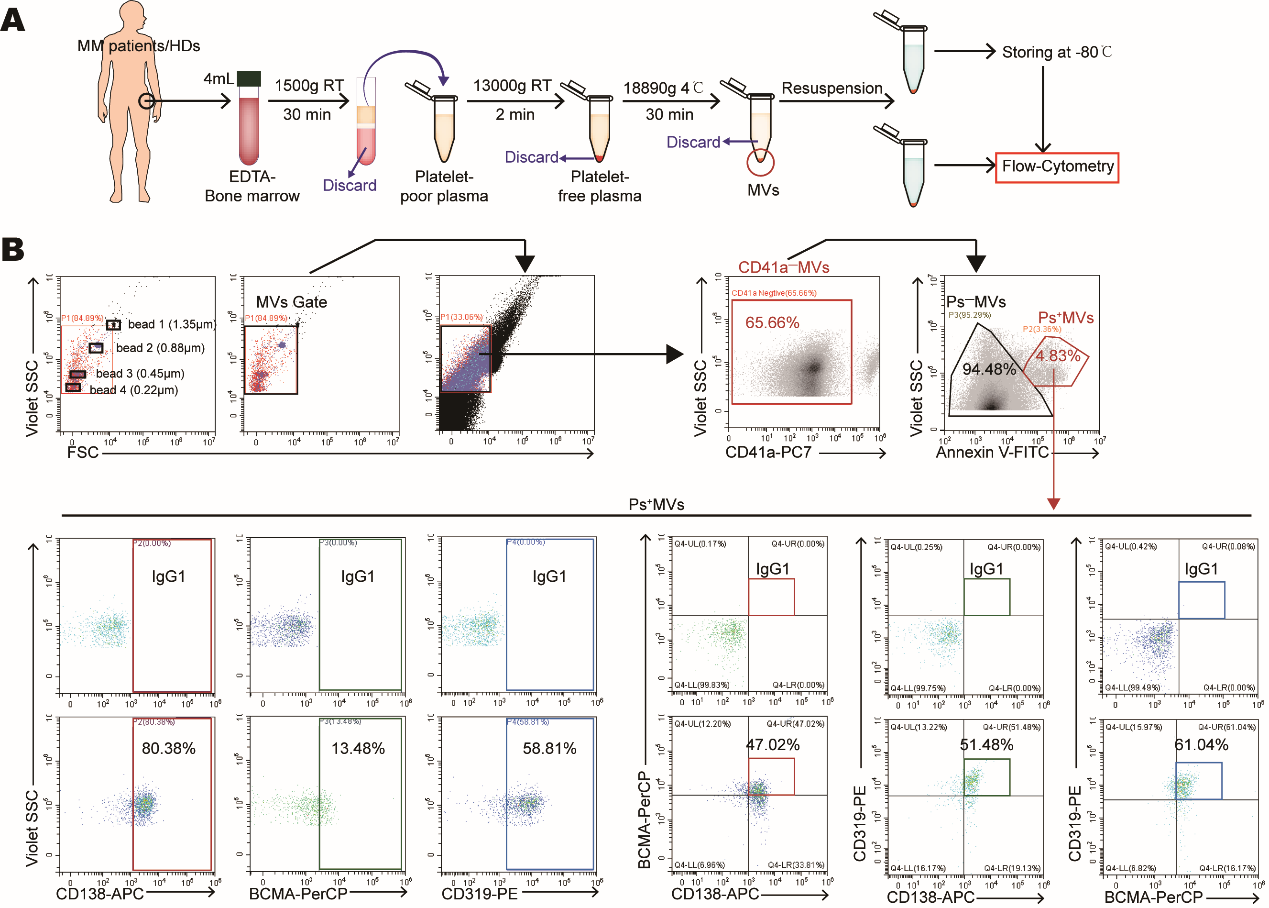


The strategy for analyzing microvesicles via flow cytometry. **A** Isolating microvesicles by differential ultracentrifugation from bone marrow. **B** Using the standardized diameter latex beads (0.22um, 0.45um, 0.88um, and 1.35um) to identify the “MVs Gate”, CD41a-PC7 was used as a marker to remove the platelet-derived microvesicles and obtain CD41a^−^ microvesicles (65.66%), and Annexin V-FITC was used to distinguish Ps^+^ microvesicles (4.83%) and Ps^−^ microvesicles (94.48%) in CD41a^−^ microvesicles. Then CD41a^−^Ps^+^ microvesicles were set as the parent groups, and CD138^+^, BCMA^+^, and CD319^+^ microvesicles were analyzed. CD138^+^BCMA^+^, CD138^+^CD319^+^, and BCMA^+^CD319^+^ microvesicles were also analyzed in CD41a^−^Ps^+^ microvesicles.

**Fig S3**


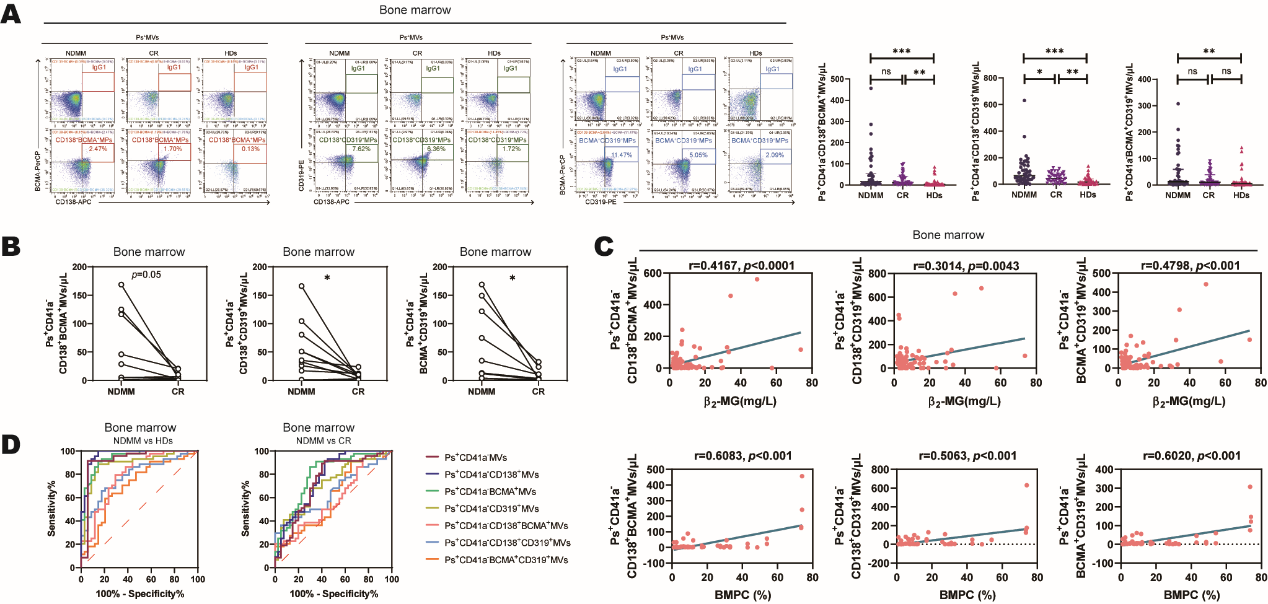


**A** The number of Ps^+^CD41a^-^CD138^+^BCMA^+^, Ps^+^CD41a^-^CD138^+^CD319^+^, Ps^+^CD41a^-^BCMA^+^CD319^+^ microvesicles from BM was significantly higher in NDMM compared with in HDs. **B** The number of BM microvesicles labeled Ps^+^CD41a^-^CD138^+^BCMA^+^, Ps^+^CD41a^-^CD138^+^CD319^+^, Ps^+^CD41a^-^BCMA^+^CD319^+^ was significantly higher in the initial treatment group (NDMM) than in the remission group (CR) after treatment. **C** The number of BM microvesicles labeled Ps^+^CD41a^-^CD138^+^BCMA^+^, Ps^+^CD41a^-^CD138^+^CD319^+^, Ps^+^CD41a^-^BCMA^+^CD319^+^ was positively correlated with the number of β2-MG and plasma cells in bone marrow smear. **D** ROC curve was analyzed Ps^+^CD41a^-^CD138^+^BCMA^+^, Ps^+^CD41a^-^CD138^+^CD319^+^, Ps^+^CD41a^-^BCMA^+^CD319^+^ microvesicles from BM to distinguish the NDMM patients from HDs (left) and CR patients (right).

**Fig S4**


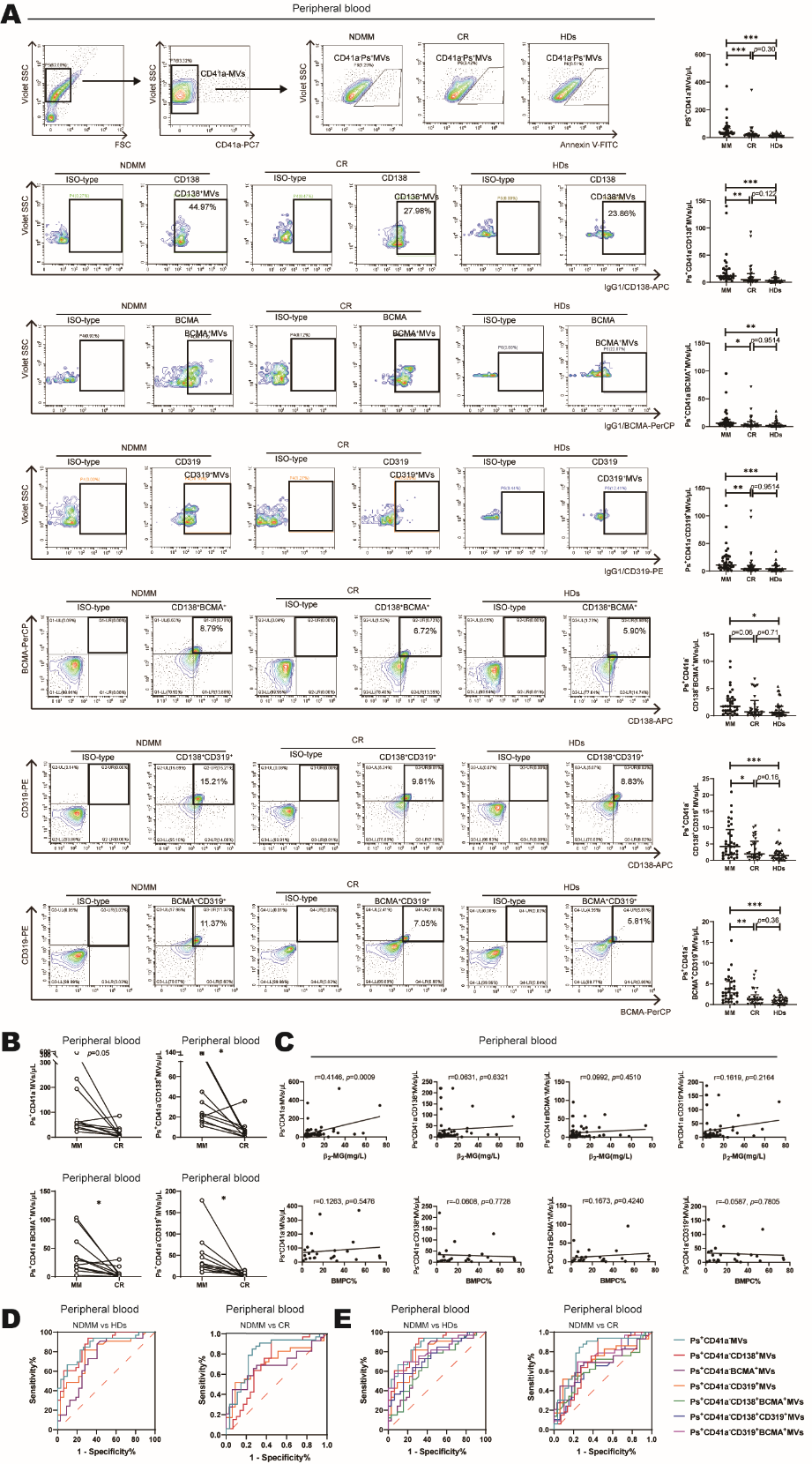


The value of microvesicles in peripheral blood in monitoring MM tumor load. **A** The number of PB microvesicles labeled CD41a^-^Ps^+^, Ps^+^CD41a^-^CD138^+^, Ps^+^CD41a^-^BCMA^+^, Ps^+^CD41a^-^CD319^+^, Ps^+^CD41a^-^CD138^+^BCMA^+^, Ps^+^CD41a^-^CD138^+^CD319^+^, and Ps^+^CD41a^-^BCMA^+^CD319^+^ in peripheral blood was significantly higher in NDMM compared with in HDs. **B** The number of CD41a^-^Ps^+^, Ps^+^CD41a^-^CD138^+^, Ps^+^CD41a^-^BCMA^+^, Ps^+^CD41a^-^CD319^+^ microvesicles from PB was significantly higher in the initial treatment group (NDMM) than in the remission group (CR) after treatment. **C** the number of CD41a^-^Ps^+^ microvesicles from PB was positively correlated with the number of β2-MG. **D** ROC curve was analyzed CD41a^-^Ps^+^, Ps^+^CD41a^-^CD138^+^, Ps^+^CD41a^-^BCMA^+^, Ps^+^CD41a^-^CD319^+^, Ps^+^CD41a^-^CD138^+^BCMA^+^, Ps^+^CD41a^-^CD138^+^CD319^+^, and Ps^+^CD41a^-^BCMA^+^CD319^+^ microvesicles from PB to distinguish the NDMM patients from HDs and CR patients.

**Fig S5**


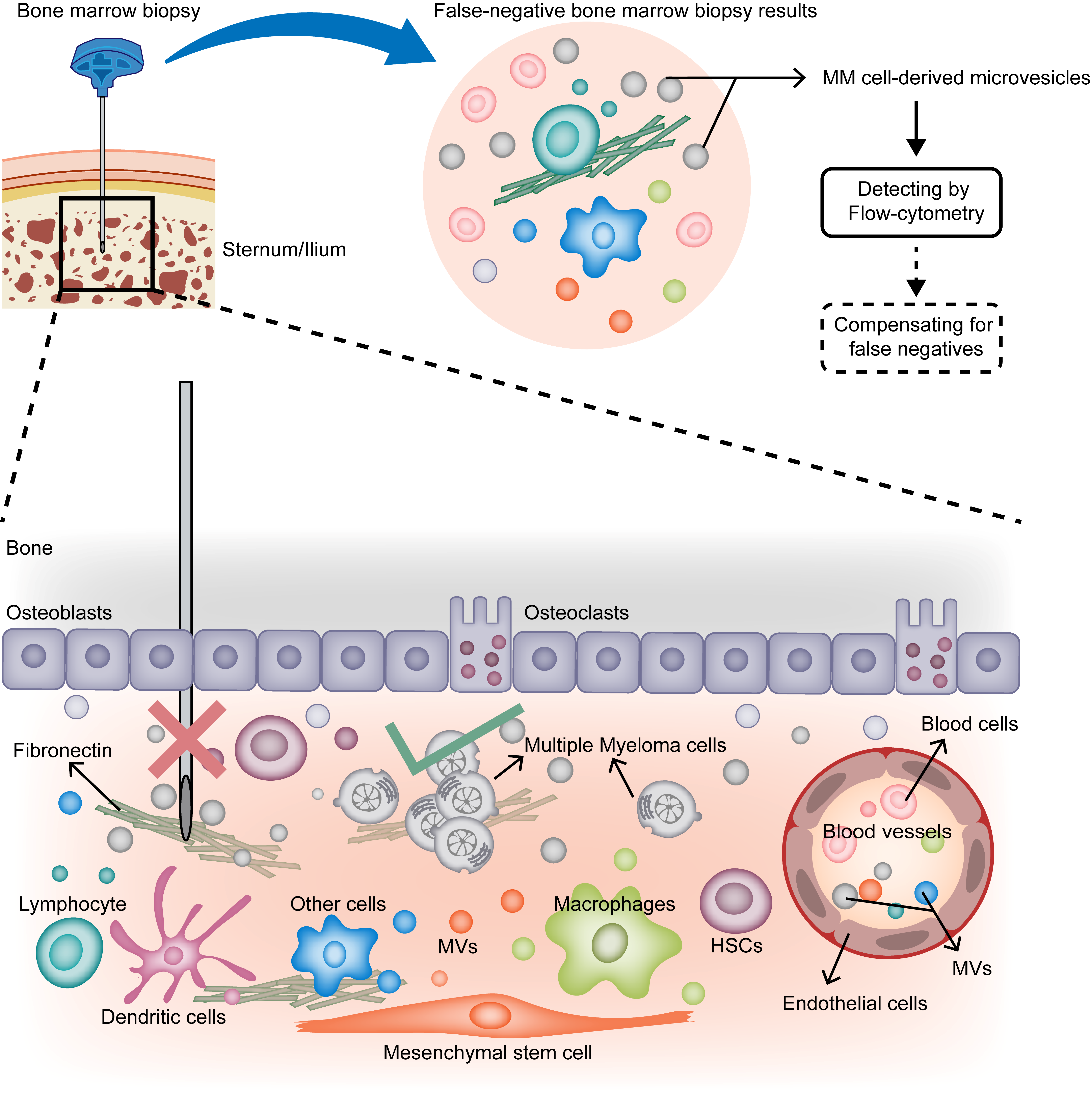


Low and even false negative results can arise due to limited volume of bone marrow aspirate specimens or unequal local distribution of bone marrow lesions, hence multi-site aspirations are frequently necessary in clinical practice. Our new liquid biopsy technique can compensate to some extent for the disadvantage.
