# Supplementary material for: Detection of myeloma cell-derived microvesicles: a tool to monitor multiple myeloma load
Source: Exp Hematol Oncol. 2023 Mar 6;12:26. doi: 10.1186/s40164-023-00392-4 (PMC9987071; doi:10.1186/s40164-023-00392-4)
Supplement: Supplementary file 2 — Additional file 2. Additional tables 1–6. [file 40164_2023_392_MOESM2_ESM.docx]

**Additional file2**

Table S1 Characteristics of the patients.

| Group | N (%) |
| --- | --- |
| Total no. of patients | 89 (100) |
| Gender (M/F) | 46/43 (52/48) |
| Age, median (Q1-Q3) | 62 (39-82) |
| Type of MM | IgG 61 (69), IgA 21 (24), IgM 2 (2), IgE 1 (1), Light chain 3 (3), nonsecretory 1 (1) |
| Stage at diagnosis (Durie-Salmon) |  |
| I | 9 (10) |
| II | 14 (16) |
| III | 66 (74) |
| Stage at diagnosis (R-ISS) |  |
| I | 9 (10) |
| II | 43 (48) |
| III | 37 (42) |
| Hb≥100g/L | 39 (44) |
| Hb<100g/L | 50 (56) |
| Ca>2.75mmol/L | 13 (15) |
| Ca≤2.75mmol/L | 76 (85) |
| Cr≥177μmmol/L | 19 (21) |
| Cr<177μmmol/L | 70 (79) |

Table S2 The number of different immunolabeled microvesicles in BM

|  | | **NDMM/ μL** | **CR/ μL** | **HDs/ μL** | **P value** |
| --- | --- | --- | --- | --- | --- |
| **N** |  | 49 | 40 | 34 |  |
| CD41a-Ps+ | / | 465.3(304.04, 624.79) | 195.3(124.82, 420.11) | 50.48(22.44, 134.28) | <0.0001 |
|  | CD138+ | 203.62(144.43, 283.46) | 96.06(43.27, 197.43) | 23.67(8.27, 59.56) | <0.0001 |
|  | BCMA+ | 58.98(24.32, 159.59) | 9.69(3.78, 36.70) | 1.215(0.405, 6.96) | <0.0001 |
|  | CD319+ | 138.34(86.18, 238.74) | 73.10(15.33, 130.35) | 14.10(3.33, 30.23) | <0.0001 |
|  | CD138+BCMA+ | 16.46(7.10, 48.62) | 11.85(4.74, 39.65) | 3.02(0.52, 8.00) | <0.0001 |
|  | CD138+CD319+ | 64.47(35.15, 140.62) | 44.99(22.62, 75.50) | 17.36(4.9, 39.65) | <0.0001 |
|  | CD319+BCMA+ | 12.54(5.68，52.13) | 10.78(3.21, 43.54) | 4.77(1.28, 11.42) | 0.0115 |

Table S3 The number of different immunolabeled microvesicles in PB

|  |  | NDMM/μL | CR/μL | HDs/μL | P value |
| --- | --- | --- | --- | --- | --- |
| N | | 33 | 29 | 32 |  |
| CD41a-Ps+ | **/** | 38.53(26.67, 77.22) | 18.95(12.77, 24.79) | 16.60(10.66, 28.59) | <0.001 |
|  | **CD138+** | 11.73(8.17, 23.14) | 4.79(2.78, 14.86) | 2.30(1.64, 3.88) | <0.001 |
|  | **BCMA+** | 6.10(3.58, 12.68) | 2.98(0.36, 7.09) | 0.46(0.08, 1.61) | <0.001 |
|  | **CD319+** | 11.06(5.83, 26.45) | 3.92(1.46, 9.28) | 0.82(0.25, 2.65) | <0.001 |
|  | **CD138+BCMA+** | 2.46(0.10, 3.62) | 0.85(0.74, 2.65) | 0.05(0.02, 1.43) | <0.001 |
|  | **CD138+CD319+** | 2.47(1.15, 4.62) | 0.99(0.62, 2.50) | 0.36(0.28, 1.83) | <0.0005 |
|  | **CD319+BCMA+** | 2.83(1.72, 6.82) | 1.24(0.48, 2.14) | 1.045(0.39, 2.12) | <0.0005 |

Table S4 The number of microvesicles from BM of MRD (+) patients and MRD (-) patients

|  | | **MRD** | |
| --- | --- | --- | --- |
|  |  | **<0.001%** | **≥0.001%** |
| **N** | | **13** | **37** |
| **Ps+** | **/** | **6.720 (1.785, 18.13)** | **66.22 (27.89, 149.5)** |
|  | **CD138+** | **1.480 (0.9250, 12.85)** | **29.61 (10.07, 61.25)** |
|  | **BCMA+** | **1.030 (0.4450, 7.450)** | **15.55 (6.458, 34.94)** |
|  | **CD319+** | **1.230 (0.6150, 4.440)** | **19.88 (5.208, 51.04)** |

Table S5 The parameters of TEM

| Instrument Model | JEM-1200EX |
| --- | --- |
| Acceleration voltage | 100KV |
| TEM point resolution | 1.60nm |

Table S6 Lasers and Filters Configuration

| Beckman Coulter CytoFLEX | | |
| --- | --- | --- |
| Lasers | Filters | Channel |
| 405nm | 450/45 BP | SSC |
| 488nm |  | FSC |
|  | 525/40 BP | FITC |
|  | 585/42 BP | PE |
|  | 690/50 BP | PerCP-Cy5.5 |
|  | 780/60 BP | PC7 |
| 638nm | 660/10 BP | APC |
