# Supplementary material for: Detection of myeloma cell-derived microvesicles: a tool to monitor multiple myeloma load
Source: Exp Hematol Oncol. 2023 Mar 6;12:26. doi: 10.1186/s40164-023-00392-4 (PMC9987071; doi:10.1186/s40164-023-00392-4)
Supplement: Supplementary file 3 — Additional file 3. Materials and methods. [file 40164_2023_392_MOESM3_ESM.docx]

**Additional file3**

**Materials and Methods**

2.1 Antibodies and reagents

The antibodies and reagents used here included: Annexin V-fluorescein isothiocyanate (BD Horizon, cat no. 51-65874X), anti-CD138-APC (clone: 44F9, lot no. 5190701751), anti-BCMA-Percp/Cyanine5.5 (clone: 19F2, lot no. 13288964), anti-CD319-PE (clone: REA150; lot no.5190924560), anti-CD41a-PE-Vio770 (clone: HIP8; lot no. 5190924444). The latex beads with a diameter of 0.22, 0.45, 0.88, and 1.35 um (cat no. NFPPS-52-4K) were all purchased from Spherotech, Germany.

2.2 Study design and patient selection criteria

Samples were collected from MM patients and healthy donors (HDs) (>18 years of age). HDs were age-matched with patients who presented normal hematology at the hospital. Pregnant candidates were excluded from this study. In total, 34 HDs and 89 MM patients were assessed, including newly diagnosed multiple myeloma (NDMM) (n = 49) and CR (n = 40). The gender (male/female) of MM patients was 46/43. Patients with CR are defined via two consecutive assessments’ demonstrating negative serum and urine immunofixation electrophoresis, with <5% bone marrow plasma cells. Certain patients’ results, based only on serum free light chain, were accepted upon their return to normal free light chain ratios.

2.3 Isolation of microparticles from the bone marrow

Up to 4 mL of Ethylenediaminetetraacetic acid (EDTA) bone marrow was centrifuged at 1500×*g* for 30 min at room temperature (RT). The precipitate was discarded to obtain platelet-poor plasma, followed by centrifugation at 13,000×*g* for 2 min at RT to obtain platelet-free plasma (PFP) from the supernatant. The PFP was divided into 200μL aliquots, which were subjected to microparticle isolation by ultracentrifugation at 18890×*g* at 4 °C for 30 min **[1, 2]**. Immediate sample analysis or storage was conducted at -80°C until analysis. All the frozen samples were thawed on ice before immunolabelling. Then the supernatant was removed, and the MP pellet was immunolabeled for flow cytometry. Technical triplicates were performed for each patient’s microvesicles count to reduce error.

2.4 Flow cytometry

All the phenotyping and counting of microvesicles were performed using a Cyto-Flex flow cytometer (Beckman Coulter, USA). An MP gate was set on the 2D scatter plot of FSC-A vs. Violet SSC-A. Latex beads of 0.22um, 0.45um, 0.88um, and 1.35um in diameter respectively, were used to define the range of MP gate. Compared to latex beads, microvesicles have lower refractive indices and their diameter is too small to be detected using conventional methods, therefore, we adjusted the side scatter (SSC) to 405nm (instead of 488nm) laser light to improve sensitivity of MP detection and avoid background noise during acquisition **[3]**. This predefined MP gate was applied to all samples. CD41a^−^, Annexin V^+/−^, CD138^+/−^, BCMA^+/−^, and CD319^+/−^ were applied to MP populations. All fluorescence positivity gates were defined based on the background fluorescence in isotype and negative controls. Before each sample was run, the flow cytometer was washed with deionized water until the number of particles was <100/s, thus reducing interference from possible impurities. Samples were run at low flow rate (about 10-30μL/min) to reduce the occurrence of coincidence. If the sampling rate was high (particle count was >10000/s) and the dropout rate is also high, the “acquisition rate setting” was adjusted to “high”. Compensation matrices applying and data analyzing were used CytExpert Analysis Software (Beckman Coulter, USA).

2.5 Surface protein phenotyping of microparticles

CD41a, Annexin V, CD138, BCMA, and CD319 were used to immunolabel microvesicles isolated from BM of MM patients and healthy donors in parallel with relevant isotype and negative controls. Firstly, the isolated MP pellet was immunolabelled with 5μL of Annexin V-FITC, and placed in the dark for 5 min at RT. Secondly, to exclude the effect of platelet-derived microvesicles, the isolated MP pellet was labelled with 5μL of anti-CD41a-Vio770 for 30 min in the dark at RT. At the same time, using 5μL of anti-CD138-APC, 5μL of anti-BCMA- Percp/Cyanine5.5, and 5μL of anti-CD319-PE labelled MP pellet for 30 min in the dark at RT. The isotype controls were multiply labelled for 30 min in the dark at RT, using 5μL of anti-IgG1-PE-Vio770, 5μL of anti-IgG1-APC, 5μL of anti-IgG1-Percp/Cyanine5.5, and 5μL of anti-IgG1-PE. While the negative controls were only labelled by 5μL of Annexin V-FITC. Thirdly, the 10×Bunding buffer was diluted to 1× at 1:9, then 200μL of diluted buffer was add into per 100μL labeled sample. This step is indispensable because the Calcium (Ca^2+^) dependence of Annexin V.

2.6 Cells and cultures

The human MM cell line, RPMI-8226 (NCI-DTP Cat# RPMI-8226, RRID:CVCL_0014), was purchased from the Peking Union Cell Bank (Beijing, China). Cells were cultured in a humidified atmosphere (37.5 °C, 5% CO_2_) using RPMI 1640 medium (Boehringer, Ingelheim, Germany) containing 10% foetal calf serum (FBS, Gibco, California, USA) and 100ug/mL penicillin (Gibco) and 100U/ml streptomycin (Gibco). The foetal calf serum was centrifuged at 120000×*g* at 4℃ for 18 hours to remove the microvesicles via Ultracentrifuge (Optima XE, Beckman Coulter, USA).

2.7 Western blotting

Western blot analysis was performed to evaluate the MLC/p-MLC protein concentrations in the RPMI-8226 supplemented with Pim-2 inhibitor-Smi16a. The RPMI-8226 cells were lysed on ice with a lysis buffer [phenylmethylsulfonyl fluoride protease inhibitor (Sigma-Aldrich), phosphatase inhibitor (Cell Signaling Technology, Danvers, MA, USA), and RIPA lysis buffer (Sigma-Aldrich) configured in a proportion of 1:1:100]. Then, protein concentrations were determined via a bicinchoninic acid (BCA) protein assay kit (Dingguo Changsheng, Beijing, China). The proteins (30ug/lane) were separated using an 8% gels SDS–polyacrylamide gel electrophoresis (SDS-PAGE). The separated proteins were transferred to a polyvinylidene diﬂuoride (PVDF) membrane (Solarbio, Beijing, China). PVDF membranes were blocked with a solution containing 5% skim milk and incubated overnight at 4 °C using the following antibodies: myosin light chain-2 (Abcam, Cambridge, MA, USA), P-myosin light chain-2 (Abcam, Cambridge, MA, USA) and β-Tubulin (Cell Signaling Technology, USA). Above antibodies were diluted at 1:1000 in 5% bovine serum albumin (BSA). The PVDF membranes were washed three times with Tris-buffered saline [0.1% Tween-20 (TBST, Solarbio, Beijing, China)], incubated for 1 h at RT with horseradish peroxidase conjugated anti-rabbit IgG sheep antibody (1:5000 dilution in 5% BSA; Cell Signaling Technology, Danvers, MA, USA), and washed another three times with TBST. The protein bands were visualized using a chemiluminescence kit (Solarbio, Beijing, China).

2.9 Statistical analysis

Statistical analyses were conducted using IBM SPSS Statistical version 20.0 for Windows (IBM SPSS Software, RRID: SCR_002865, Chicago, IL, USA, www.ibm.com). Statistical graphs were drawn using GraphPad Prism version 8.0.2 for Windows (GraphPad Software, RRID: SCR_002798, San Diego, California, USA, www.graphpad.com). The Shapiro−Wilk test was used to assess the distribution normality of data for each group. The Mann-Whitney U test was used for nonparametric data, and the data were presented as the median (Q1, Q3), and Mann-Whitney constant U and *p* values were stated. The paired t-test was used to analyze the significance between the matching data. The Spearman method was used for the correlation analysis. The relative gray scale of MLC/p-MLC/β-Tubulin protein expressions were expressed as the mean ± standard deviation (SD). All the data were expressed as numerical variables. The results were considered statistically significant at predictive values of ****p*<0.001, ***p*<0.01, and **p*<0.05.

**Refence**

1. Magnette A, Chatelain M, Chatelain B, Ten Cate H, Mullier F. Pre-analytical issues in the haemostasis laboratory: guidance for the clinical laboratories. Thrombosis journal. 2016; 14: 49.
2. Krishnan SR, Luk F, Brown RD, Suen H, Kwan Y, Bebawy M. Isolation of Human CD138(+) Microparticles from the Plasma of Patients with Multiple Myeloma. Neoplasia. 2016; 18: 25-32.
3. McVey MJ, Spring CM, Kuebler WM. Improved resolution in extracellular vesicle populations using 405 instead of 488 nm side scatter. Journal of extracellular vesicles. 2018; 7: 1454776.
